# Latent network-based representations for large-scale gene expression data analysis

**DOI:** 10.1186/s12859-018-2481-y

**Published:** 2019-02-04

**Authors:** Wajdi Dhifli, Julia Puig, Aurélien Dispot, Mohamed Elati

**Affiliations:** 1 University of Lille, 42, rue Paul Duez, Lille, 59000 France; 2 UMR 8030 ; Génomique Métabolique / Laboratoire iSSB ; CEA-CNRS-UEVE, Genopole campus 1, 5 rue Henri Desbruères, Évry, 91030 Cedex France

**Keywords:** Latent signals, Network-based transformations, Gene expression, Gene perturbation, Regulator activity

## Abstract

**Background:**

With the recent advancements in high-throughput experimental procedures, biologists are gathering huge quantities of data. A main priority in bioinformatics and computational biology is to provide system level analytical tools capable of meeting an ever-growing production of high-throughput biological data while taking into account its biological context. In gene expression data analysis, genes have widely been considered as independent components. However, a systemic view shows that they act synergistically in living cells, forming functional complexes and more generally a biological system.

**Results:**

In this paper, we propose LatNet, a signal transformation framework that, starting from an initial large-scale gene expression data, allows to generate new representations based on latent network-based relationships between the genes. LatNet aims to leverage system level relations between the genes as an underlying hidden structure to derive the new transformed latent signals. We present a concrete implementation of our framework, based on a gene regulatory network structure and two signal transformation approaches, to quantify latent network-based activity of regulators, as well as gene perturbation signals. The new gene/regulator signals are at the level of each sample of the input data and, thus, could directly be used instead of the initial expression signals for major bioinformatics analysis, including diagnosis and personalized medicine.

**Conclusion:**

Multiple patterns could be hidden or weakly observed in expression data. LatNet helps in uncovering latent signals that could emphasize hidden patterns based on the relations between the genes and, thus, enhancing the performance of gene expression-based analysis algorithms. We use LatNet for the analysis of real-world gene expression data of bladder cancer and we show the efficiency of our transformation framework as compared to using the initial expression data.

## Background

The last few years have seen major advancements in experimental procedures, making it possible to gather huge quantities of biological data. Scientists now routinely measure, characterize and localize an ever-growing number of molecules at the level of entire biological systems. However, despite the continuous expansion of omics approaches contributing to the elucidation of systems-level networks, we still know little about the organization of discrete biological activities in space and time, and their integration into larger systems and coherent phenotypes. The main difficulty lies in bridging the growing gap between high-throughput biological data production and analytical tools capable of developing a system level view of the data that also takes into account its biological context. Gene expression data analysis has become one of the most active fields in bioinformatics and computational biology. Although genes have been considered as independent components in multiple expression data-based analyses, in living cells and organisms, they act together in harmony forming functional networks and more generally a biological system. In this context, multiple inference methods of regulatory networks have been developed, and were recently reviewed in [1]. Most of them fall into the domain of machine learning or empirical inference [2] and they usually use expression data obtained from microarray or RNA-seq technology. However, regulatory network reconstruction is not the ultimate goal but an important intermediate step that addresses diverse biological and biomedical questions. Thus, novel computational approaches are still required for capturing latent biological system relationships (e.g., regulator activity, post-transcriptional control, gene perturbation, *etc*.. Linear and non-linear transformations of expression data could be derived from specific mechanistic models (*e*.*g*, regulatory networks [3, 4]) and statistical measurements (*e*.*g*, Matrix Factorization (MF) [5]), and could play a key role in capturing such indirect and latent relationships. Principal Component Analysis (PCA) [6], Singular Value Decomposition (SVD) [6] and Non Negative Matrix Factorization (NMF) [7] are among the most widely used state-of the-art MF methods for extracting latent variables from an input signal through a data decomposition. MF was first applied to gene expression data analysis in the early 2000s [8, 9] with broad successful applications to unsupervised clustering, component identification, and prediction [5]. The main drawback with MF approaches is that they suffer a difficulty in the interpretability of the resulting factorized components. This has imposed a serious focus on the analysis of these components in the form of metagenes and metasamples, to facilitate their interpretability and association to biologically relevant mechanisms [9, 10].

In this paper, we propose a generic network-based transformation framework that allows to generate from an initial expression data new latent representations based on a network of relations between the genes. Our approach aims to leverage the background knowledge about the underlying hidden structure of the biological system between genes that could be derived from gene regulatory networks. In contrast to MF methods, our approach generates latent signals that are associated to regulators and genes of the network and thus biological interpretations could be performed directly on the output signals. This approach has also a direct impact on many important algorithms (for visualization, classification, clustering and more) which are at the heart of major bioinformatics applications including diagnosis and personalized medicine. Indeed, most of these algorithms could perform poorly when the used gene expression signal of the genes (as features) is noisy or not informative for the considered task. By using a network structure to transform the initial expression data, our framework will help in uncovering latent signals that could emphasize hidden patterns based on the relations between the features and thus enhancing the performance of these algorithms. Concretely, our transformation framework takes as input a gene expression dataset and a regulatory network expressing the relations between the regulatory elements (mainly transcription factors and miRNA) and their target genes. The new transformed values are at the level of each sample/condition and thus could be directly used instead of the initial expression data, for instance, for the classification of cancer subtypes. We use our framework for the analysis of real-world gene expression data of bladder and breast cancer and we show the efficiency of our transformation framework as compared to using the initial expression data as well as other state-of-the-art approaches for extracting latent features.

## Methods

Genes have been considered as independent components (features) in multiple expression data-based analyses [11–13]. However, in real-world biological organisms, they interact together in a systemic way to ensure the consistency of the functional machinery of the cell. Considering these relations between the genes is very important for statistical gene expression-based analyses as it allows to boost their accuracy by making the analyses better reflect the underlying mechanisms of the biological system under study. Yet, these relations have not been sufficiently exploited in the literature. In fact, the relations between genes could be expressed through a network structure where the nodes could be the genes and the edges express their relations. Multiple network representations are possible in this context and have shown to be very informative, including co-expression [14], regulatory [15], co-regulation [16] and co-regulatory networks [3]. For instance, in [16], the authors proposed a method for clustering genes using a network of co-regulations that is derived from an input regulatory network, where genes are represented by graph nodes and an edge connects two nodes if they share a high number of regulators. The authors showed that this approach allows to discover modules that are highly enriched in terms of gene ontology (GO) associations and that are not captured by classical clustering techniques.

**Fig. 1:**
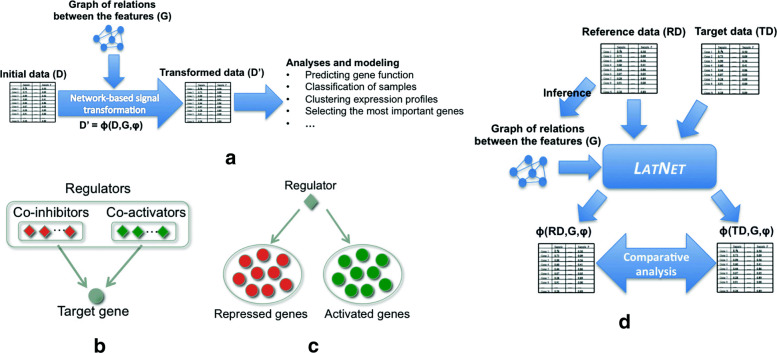
General framework. (**a**) An overview of our framework LATNET for latent network-based representations. (**b**) A regulatory program (network) of a target gene with its co-activators and co-inhibitors. (**c**) An example of a regulator and its set of activated and repressed genes. (**d**) An example of a pipeline for comparative analyses based on LATNET transformed signals

We propose a generic framework that could leverage any network structure that defines relations between the features and appropriate measurements for expression signal transformation into novel representations to unravel hidden patterns. Figure 1a shows an overview of our framework (termed LATNET for LATent NETwork-based representations). The signal transformation schema in our framework could be formalized as follows. Given an input expression dataset *D* of *n* samples/conditions, let *Ω* be the set of *m* features (genes) defined over the samples. Let *G* be a network structure that defines the relations *E* between the features (*G*=(*Ω*,*E*)) and *ϕ* is a measure defined over the expression values of the features in *Ω* based on their relations in *G*. We define *Φ* as the transformation function that uses *ϕ* and *G* to derive a new representation *D*^′^ of *D* based on the elementary transformations of *ϕ* such that *D*^′^=*Φ*(*D*,*G*,*ϕ*). In the following, we present a concrete implementation of LATNET based on the LICORN [15] network structure and two novel network-based signal transformation techniques of input expression signals. The first transformation technique operates at the level of target genes to capture gene perturbation signals and the second one estimates the activity of network regulators in a given set of expression conditions. Note that LATNET does not depend on the platform where the data comes from, and thus could be used on any kind of large-scale gene expression data such as microarray and RNA-Seq data.

### Gene regulatory network as an underlying structure between the features

In this work, we use gene regulatory networks (GRNs) as the structure that defines the directed connections between genes. GRNs also define an informative hierarchy between genes that puts transcription factors at the top level and target genes at the bottom level in the form of a bipartite graph. The inference of GRNs has been extensively studied in the literature and a large number of free tools are available for it [1], making the acquirement of such a network very easy. Besides, for many organisms, a large number of transcription factors (TFs), genes and regulatory interactions have been experimentally validated and are available in online databases (e.g. TRRUST [17]: TF–target interaction database for humans). We propose to consider a GRN as a background network structure that defines the relations between genes (used as features in gene expression-based data analyses) and to exploit this structure to perform a transformation of the input signal of expression for unravelling latent signals that are more informative than the initial expression data. In this study, we use LICORN [15, 18] approach for the inference of regulatory networks. LICORN identifies groups of regulators as co-activators *A* and co-inhibitors *I* for each target gene. We formalize a local regulatory network as *GRN*(*g*)=(*A*_*g*_,*I*_*g*_), and the global regulatory network as a graph *G* that is defined by *G*=(*V*,*E*) such that *V*=*V*^*R*^∪*V*^*T*^, where *V*^*R*^ is the set of regulator nodes, *V*^*T*^ are the target nodes and *E* is the set of regulatory connections (the edges). We also note that for a target gene *g*, $V^{R}_{g}=A_{g}\cup I_{g}$.

### Network-based quantification of sample-specific gene perturbation

In this section, we present our first data transformation technique that allows to capture latent perturbation signals for each gene at each sample by taking into account a local regulatory program (network) that defines the baseline state of the expected regulations. We introduce a model based on a regulatory process (see Fig. 1b), allowing genes not to respond to their regulators in the expected manner, i.e., to be perturbed. This approach models the LICORN inferred GRN structure (∀*g*∈*V*^*T*^,*GRN*(*g*)=(*A*_*g*_,*I*_*g*_)) by combining, for each gene, its set of co-activators *A*_*g*_ and co-inhibitors *I*_*g*_ in a regression model that estimates the expression level of the target gene ($\widehat {y}$). The regression model is defined as follows:

1 $$ \widehat{y}=\sum\limits^{q+p}_{j=1}\alpha_{j}*r_{j} + \alpha_{a}\prod\limits^{q}_{k=1}a_{k} + \alpha_{i}\prod\limits^{p}_{l=1}i_{l} +\beta,  $$

where *q* and *p* are the numbers of co-activators (*q* = |*A*_*g*_|) and co-inhibitors (*p* = |*I*_*g*_|), *r*_*j*_ is the expression of the *j*^*th*^ regulator in $V^{R}_{g}$, *a*_*k*_ is that of the *k*^*th*^ activator in *A*_*g*_, and *i*_*l*_ is that of the *l*^*th*^ inhibitor in *I*_*g*_. Note that the last variables (i.e., *a*_*k*_ and *i*_*l*_) are inserted in the regression model in order to promote the cooperativity mode of activators and inhibitors [15, 18].

This regression model is optimized using a least-squares estimation:

2 $$ \forall g \in V^{T}, \hspace{0.5cm} \widehat{\alpha} = {\underset{\alpha}{\arg\min}} \sum\limits_{i = 1}^{m}(\widehat{y}_{i} - y_{i})^{2},  $$

where $\widehat {\alpha }$ is the vector of *optimal* regression coefficients for the network regulatory model of gene *g*, $\widehat {y}_{i}$ is the expected expression level of *g* in sample *i*, *y*_*i*_ is its actual expression value of *g* in sample *i* and *m* is the number of samples in the dataset. Note that Eq. 2 is optimized on a reference dataset, which is typically the one on which the network models were constructed.

Now, given a calibrated reference model and a query expression dataset (abnormal/stress/disease conditions), the perturbation level for each gene is computed based on the expected expression and the observed one. In other words, we use the reference network-regression model to estimate the expression of the target gene given the expression of its regulators. Then, we compare the observed expression level with the expected one, to capture significant unexpected changes of high over/under-expression. One would expect unperturbed genes to respond to their regulators in the expected manner as expressed by the network regression model that was fitted on reference samples. The estimated level of perturbation for a target gene *g*∈*V*^*T*^ is simply computed as:

3 $$ \widehat{Pert}(g) = \widehat{y} - y = \sum\limits^{q+p}_{j=1}\alpha_{j}*r_{j} + \alpha_{a}\prod\limits^{q}_{k=1}a_{k} + \alpha_{i}\prod\limits^{p}_{l=1}i_{l} +\beta - y,  $$

where *y* is the expression of *g* in the target sample and $\widehat {y}$ is its estimated expression based on its fitted reference regulatory regression model and the expression of its regulators in the target sample. Note that the type of the perturbation (i.e., under or over-expression) could be captured from the sign of the raw difference between the estimated and the observed expression $(\widehat {y} - y)$ and that it is possible to use the absolute operator to flatten positive and negative perturbation differences into a distance level. We also emphasize that the proposed formalization allows to estimate per sample perturbation levels which could be of interest in multiple applications where the analysis and/or the decision making is personalized and sample specific. It is important here to clarify that it is possible to leverage existing differential expression (DE) analysis measures [11, 13] in this framework. Yet, the proposed approach differs from existing DE techniques in fundamental key aspects, in the sense that, here, we aim to unravel latent perturbation signals and not differentially expressed genes. Unlike DE techniques that focus on the direct comparison of the expression of genes in different conditions, here, we make use of the underlying structure of regulatory networks of genes and we associate regulatory weights to each single regulator as well as to the groups of co-activators and co-inhibitors, through a statistical linear model. The associated weights reflect the estimated true regulatory power that each member of the model has on the calibration of the target gene expression.

### Network-based quantification of sample-specific regulator activity

In this section, we present our second transformation technique, that allows to capture latent signals of the regulators activity at each sample by taking into account the regulatory network structure that defines, for each regulator, its set of activated and repressed genes in baseline reference conditions. For this purpose, we introduce a model based on a regulatory process (see Fig. 1c) allowing to capture the true activity level of a regulator, not based on its own expression level, but on its observed effect on downstream entities. This approach also models the LICORN-inferred GRN structure by comparing, for each regulator *r*, the distribution of its activated *A*^*r*^ and repressed *I*^*r*^ genes (∀*r*∈*V*^*R*^,*targets*(*r*)=(*A*^*r*^,*I*^*r*^)). This model is based on the work in [3], where the *influence* measure was introduced to estimate the activity of a regulator through a Welch t-test by comparing the distribution of the expression of *A*^*r*^ and *I*^*r*^. The *influence* of a regulator *r* is computed as follows:

4 $$ Influence(r) = \frac{\overline{E\left({A^{r}}\right)}-\overline{E\left({I^{r}}\right)}}{\sqrt{\frac{{\mu^{2}_{A_{r}}}}{\left|{A_{r}}\right|} + \frac{{\mu^{2}_{I_{r}}}}{\left|{I_{r}}\right|}}},  $$

where *E*(*A*^*r*^) and *E*(*I*^*r*^) are respectively the expressions set of the activated and repressed genes in the samples. $\overline {E\left (A^{r}\right)}$ and $\overline {E\left (I^{r}\right)}$ are their respective means and $\mu ^{2}_{A_{r}}$ and $\mu ^{2}_{I_{r}}$ are their standard deviations.

We propose an adaptation of the *Influence* measure as follows:

5 $$ Activity(r) =\left\{\begin{array}{ll} Influence(r), & \; if \; Influence(r)>0\\ 0, & \; otherwise.\\ \end{array}\right.  $$

Unlike the *Influence* measure, *Activity* considers that the regulator is active only when it activates *A*^*r*^ and represses *I*^*r*^ as expected by the network reference model, which is reflected by a positive welch t-test value. The regulator is more active when this value is higher. We consider that negative values of the test do reflect the absence of activity of the regulator (*Activity*(*r*)=0) and we do not attribute them to the latter.

### Scalability of LATNET to large datasets

With the recent advancements in high-throughput experimental procedures, biologists are gathering huge quantities of data in a fast pace. For a practical usage of computational analysis tools, it is important that they be capable of efficiently handling large scale inputs to meet the ever-growing production of data. We conceived LATNET in a way that allows it to leverage parallel and distributed computational resources. For both network transformations (i.e., regulator activity and gene perturbation) the computation is performed independently for each regulator or target gene and on each sample. Thus, each computation could be run in a single process in parallel/distributed architectures (e.g., multi-core, cloud computing) making LATNET capable of efficiently handling extremely large datasets.

### Usefulness of LATNET in bioinformatics applications

In this section, we present potential bioinformatics applications that are based on expression data and that show the usefulness of our framework.

**Clustering.** Clustering is an exploratory task that aims to capture groups of homogeneous objects based on their similarities. Capturing similarities between the data instances is the core task of all clustering methods. Clearly, the robustness of this task is highly related to the quality of the signals in the input data. In this context, latent activity/perturbation signals could help capturing different regularities in the data and thus emphasizing clusters of interest with similarities of cohesive latent signals that are difficult to capture directly from the input expression data.

**Classification.** Another very important application of LATNET is classification where the aim is to predict the class label of an unknown object based on a reference set of objects with known labels. Similarly to clustering, the performance of a classifier could be impacted by the quality of the input data. The latent signals derived by our framework could also be used to improve the classification in multiple expression data-based methods. For instance, these latent signals can be used as robust clinical biomarkers or tumor-type specific transcriptomic signatures of tumoral cells.

**Comparative analyses.** Multiple gene expression based studies rely on comparative analyses where the expression of genes is compared across multiple samples of different experiments. LATNET could also be used in this context, for instance, for ranking regulators based on their activities to unravel master regulators in the system or for ranking genes according to their perturbation signals to capture perturbed ones. The same experiments could also be performed between multiple expression datasets for comparative analyses (e.g., different stress conditions or different subtypes of a disease) to capture context specific markers. Figure 1d shows an example of a pipeline for comparative analyses using LATNET.

## Experimental data and settings

### Experimental dataset

To empirically assess the efficiency of LATNET, we perform a study on three gene expression datasets deposited at the ArrayExpress [19] or Gene Expression Omnibus [20] databases through accession numbers: E-MTAB-1803 [21], E-TABM-147 [22] and GSE32894 [23]. All three datasets consist of gene expression profiles of human bladder cancer patients. For each tumor sample, gene expressions are available from experimental data, and the samples are classified into two classes according to whether the cancer is muscle-INVASIVE or SUPERFICIAL. The datasets characteristics are reported in Table 1.

**Table 1:** Number of genes, samples and classification of the bladder cancer datasets

	Genes	Tumor samples	Invasive	Superficial
E-MTAB-1803	20,326	193	89	104
E-TABM-147	8174	79	43	36
GSE32894	15,092	306	93	213

### Network inference

In order to obtain a graph structure that presents the underlying relations between the features (genes) of a transcriptomic dataset, we use LICORN [15, 18] (available in the COREGNET Bioconductor R package [3]). LICORN is a data mining algorithm that allows the inference of gene regulatory networks that can capture the targets of transcription factors from genome wide expression data. Note that LATNET does not depend on the network inference method and can also leverage regulatory networks from any other inference methods, such as ARACNE [24] or CLR [25]. Note also that the evaluation of the used network inference method has previously been addressed in [3, 15, 18] and is beyond the scope of this paper. For this study, we inferred the regulatory networks using LICORN with its default input parameters.

### Generation of latent transformations

We apply LATNET on the three selected bladder cancer gene expression datasets: E-MTAB-1803, E-TABM-147 and GSE32894. We denote by LATNET ^*P*^ and LATNET ^*A*^ the signals generated by LATNET. The resulting number of features for the new signal of each dataset is reported in Table 2. Note that in order to conduct a fair comparison, we restricted the EXPRESSION data exclusively to the genes present in the network, hence the decrease in the number of features compared to Table 1.

**Table 2:** Number of features in EXPRESSION, LATNET ^*A*^ and LATNET ^*P*^ data

	Number of features		
	Expression	LatNet ^*A*^	LatNet ^*P*^
E-MTAB-1803	7089	667	6359
E-TABM-147	3238	394	2773
GSE32894	5858	606	5190

To compare with existing competitors, we generate three other signals from the original gene expression data with three state-of-the-art latent methods namely PCA [6], SVD [6] and NMF [7]. We applied the PCA, SVD and NMF methods on the original gene expression data and obtained new datasets with 2, 10 and 10 features, respectively.

### Stability analysis

The ability of a method to select the same features after perturbing the dataset is a determinant factor. We apply a learning procedure (support vector machines [26]) on random subsets of samples of the original dataset to extract its associated set of selected features. The number of repetitions is set to 20, the size of the sampled subsets are 90% of the original dataset and the used feature selection approach is the Recursive Feature Elimination (RFE) [27] with the number of selected features set to 100. The stability was estimated by measuring the average overlap of all pairs of selected features on the subsamples. Formally:

6 $$ stability = \frac{2 {\sum}_{N_{s}}^{i=1} {\sum}_{N_{s}}^{j=i+1} F\left(f_{i},f_{j} \right)}{N_{s}\left(N_{s} -1\right)}  $$

where *f*_*i*_ and *f*_*j*_ represent the sets of selected features for different subsets of data, and *F* is a function that measures the overlap between the two signatures. Here, we use the Kuncheva index [28] (*Ku*) defined as:

7 $$  Ku\left(f_{i}, f_{j}\right) = \frac{\left|f_{i} \cap f_{j} \right| \cdot N - s^{2}}{s \cdot (N -s)} = \frac{\left|f_{i} \cap f_{j} \right| - \frac{s^{2}}{N}}{s- \frac{s^{2}}{N}}  $$

where *N* is the total number of features and *s*=|*f*_*i*_|=|*f*_*j*_|. Note that the Kuncheva index takes into account the bias related to the number of features in the dataset.

### Reproducibility analysis

One of the most important drawbacks of current models in genome biology is the lack of reproducibility of results when using different datasets [29]. While these methods could produce models with acceptable classification performances, they are unable to find models with overlapping attributes, a feature of high relevance in biological studies. We evaluated the reproducibility of the selected features using LATNET ^*A*^ and LATNET ^*P*^ signals on two different (comparable) bladder cancer datasets. We use SVM-RFE for feature selection on each of the two datasets and the Kuncheva index to evaluate the overlap between the sets of selected features.

## Results and discussion

### LATNET classification performance evaluation

In this experiment, we attempt to assess the quality of the two new signals (i.e., LATNET ^*P*^ and LATNET ^*A*^) on the prediction of clinical phenotypes (i.e., invasive and superficial) for bladder cancer samples of the 3 datasets (i.e., E-MTAB-1803, E-TABM-147 and GSE32894). To this aim, we use two well established classifiers namely Random Forest (RF) [30] and Support Vector Machine (SVM). All methods are applied with a 5 fold cross-validation strategy. Additionally, we reproduce the same classification task with the EXPRESSION input signal. For each of the 3 signals, we report the Area Under the Curve (AUC) obtained with both methods. Results are showed in Table 3.

**Table 3:** Classification results in terms of AUC obtained on the E-MTAB-1803, E-TABM-147 and GSE32894 datasets with Random Forest (RF) and Support Vector Machine (SVM) classifiers using input signals LATNET ^*P*^, LATNET ^*A*^ and EXPRESSION

	LatNet ^*A*^		LatNet ^*P*^		Expression	
	RF	SVM	RF	SVM	RF	SVM
E-MTAB-1803	0.93	0.91	**0.94**	0.85	**0.94**	0.93
E-TABM-147	0.88	0.87	**0.91**	**0.91**	0.90	0.83
GSE32894	0.83	0.82	0.83	0.83	0.84	**0.90**

In bold are the best AUC achieved in each of the 3 datasets

We observe that overall all the 3 input signals are comparable across the 3 bladder cancer datasets. Note that the EXPRESSION signal already produces high scores on all datasets. Thus, more in depth analyses (especially in terms of stability and reproducibility) are required for evaluation.

### Performance comparison of different latent approaches

In this section, we compare LATNET ^*P*^ and LATNET ^*A*^ to other state-of-the-art latent methods namely PCA, SVD and NMF. For the three latent signals, we repeat the classification experiment conducted previously and we report the AUC performances obtained with the RF and SVM. Table 4 shows the obtained results.

**Table 4:** Classification results in terms of AUC obtained on the E-MTAB-1803, E-TABM-147 and GSE32894 datasets with Random Forest (RF) and Support Vector Machine (SVM) classifiers using input signals LATNET ^*P*^, LATNET ^*A*^, PCA, SVD and NMF

	LatNet ^*A*^		LatNet ^*P*^		PCA		SVD		NMF	
	RF	SVM	RF	SVM	RF	SVM	RF	SVM	RF	SVM
E-MTAB-1803	0.93	0.91	**0.94**	0.85	0.93	0.87	0.93	0.90	0.92	0.92
E-TABM-147	0.88	0.87	**0.91**	**0.91**	0.87	0.87	0.86	0.90	0.84	0.89
GSE32894	**0.83**	0.82	**0.83**	**0.83**	0.75	0.75	0.77	0.81	0.82	0.82

In bold are the best AUC achieved on each dataset

We observe that although the 5 input signals generated good AUC performances across the 3 bladder cancer datasets, our approach scored best overall. We note that PCA, SVD and NMF signals present slightly lower results in the major part of the experiments with respect to LATNET ^*P*^ and LATNET ^*A*^. Moreover, we remind that the signals obtained using these approaches lack of interpretable features, whereas both LATNET signals are directly associated with the existing regulators/targets of the dataset. Thus, for the rest of the “Results” section, we exclusively use EXPRESSION as a benchmark of LATNET ^*P*^ and LATNET ^*A*^ as its classification results reported in Table 3 are more challenging for our approach.

### Stability and reproducibility of LATNET

With the activity and perturbation transformations, we do not solely aim at reducing the dimensionality of the original data, but also at providing a signal able to provide more stable and reproducible results in terms of feature selection. In order to assess the gain earned by the above mentioned transformations in terms of stability and reproducibility, we perform the following analyses. For the stability, we consider all bladder cancer datasets. For the reproducibility, we consider the E-MTAB-1803 and GSE32894 bladder cancer datasets.

**Fig. 2:**
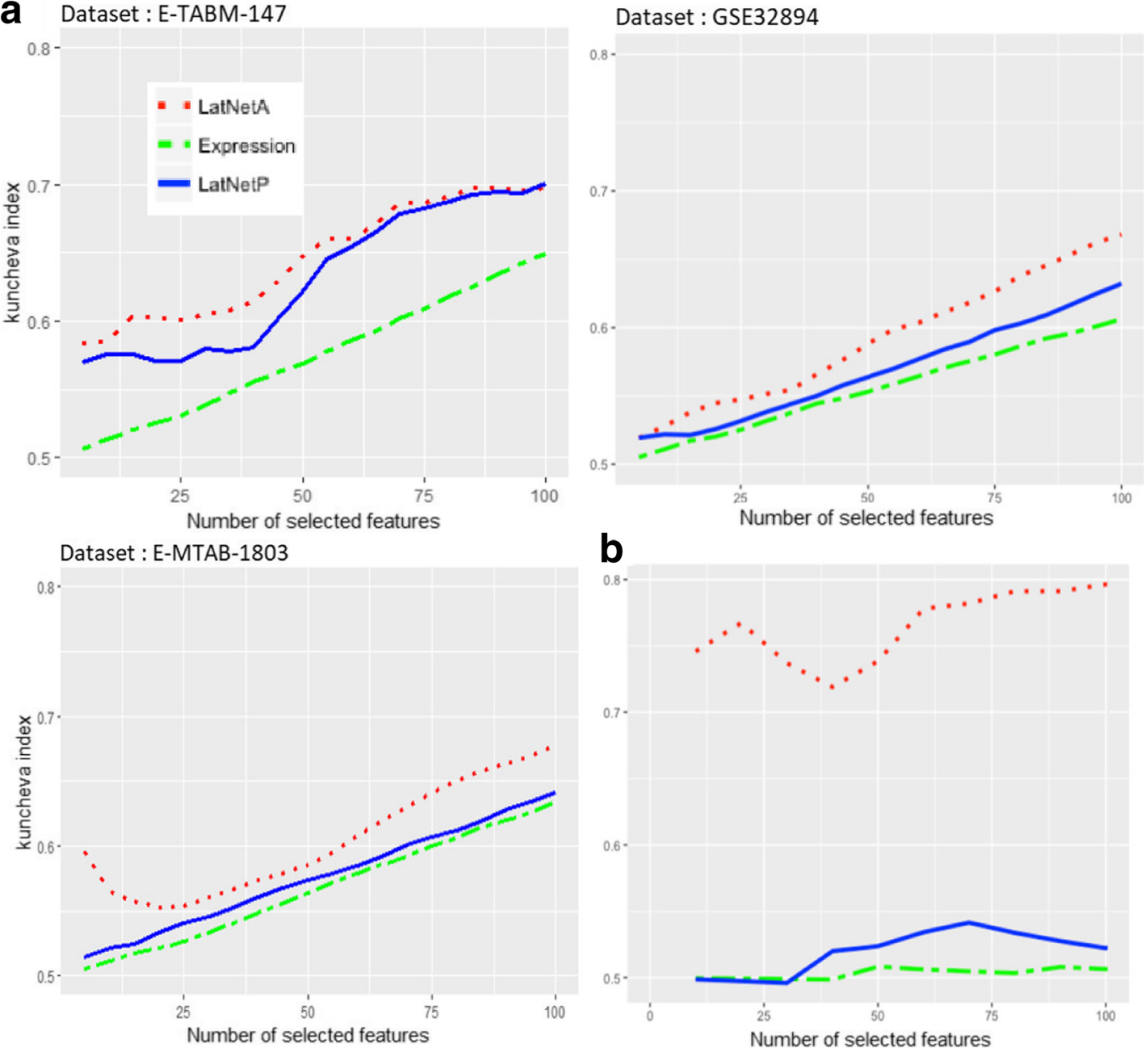
Stability and reproducibility of Expression, LATNET ^*A*^ and LATNET ^*P*^ for bladder cancer dataset. **a)** The stability of signatures depending of the number of selected features estimated by the Kuncheva index in dataset E-TABM-147, GSE32894 and E-MTAB-1803 respectively. **b)** The reproducibility between signature from GSE32894 and E-MTAB-1803 dataset. The overlap between signatures was computed by the Kuncheva index between signatures from the two dataset with the same number of selected features

The stability estimated with the Kuncheva index in either EXPRESSION, LATNET ^*A*^ or LATNET ^*P*^ for all bladder cancer datasets is presented in Fig. 2a. The stability of the feature selection method in transformed data are better with our approach and the three methods could clearly be ranked in a decreasing order of stability as LATNET ^*A*^, LATNET ^*P*^ and EXPRESSION.

Figure 2b represents the reproducibility results for EXPRESSION, LATNET ^*A*^ or LATNET ^*P*^ between two bladder cancer datasets. Concretely, we used the Kuncheva index to measure the overlap of the feature sets selected with repetitions from both GSE32894 and E-MTAB-1803 datasets. These results clearly show a much higher reproducibility with models trained on the LATNET ^*A*^ datasets, regardless of the number of selected features. Although the performances of LATNET ^*P*^ were lower than LATNET ^*A*^, it showed a higher reproducibility that EXPRESSION.

### Case study: comparative analysis and visualisation of molecular tumour subgroups

In this case study, we apply LATNET on the breast cancer BRCA-TCGA dataset which contains the expression level of 18,908 genes of 1,051 breast cancer samples. Each of the samples is classified in one of 4 molecular tumour subtypes: Basal, Her2, LumA and LumB. Note that in contrast to the bladder cancer datasets used earlier in the study, i) TCGA data is obtained from RNA-Seq and not microarray technology. ii) Moreover, molecular subtypes have been derived from gene expression itself.

**Fig. 3:**
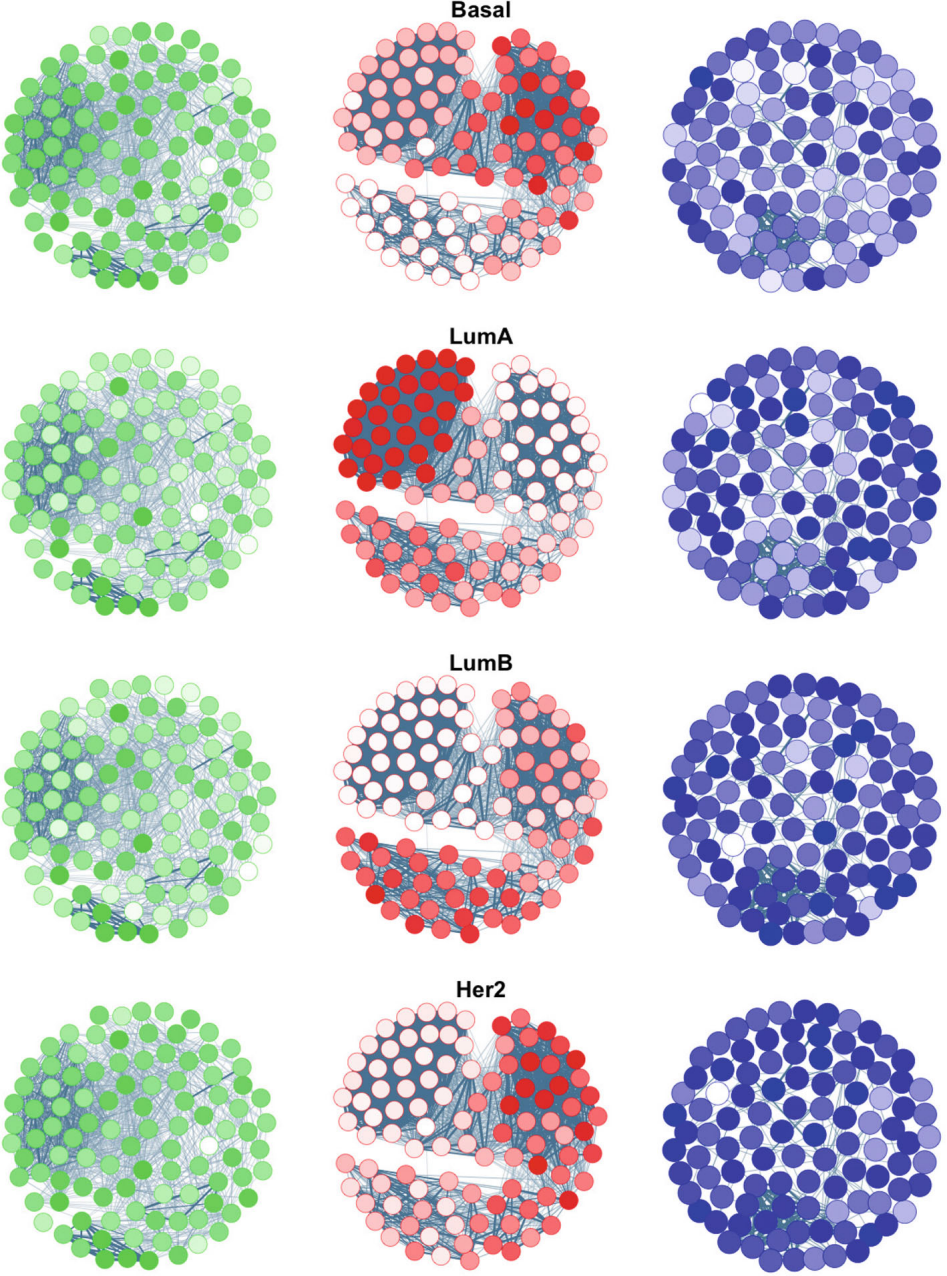
Network visualization of EXPRESSION, LATNET ^*A*^ and LATNET ^*P*^ signals (in green, red and blue, respectively) on each breast cancer subtype. Colour intensity represents the strength of the signal for the nodes

Given the BRCA transcriptomic dataset, the signals LATNET ^*A*^ and LATNET ^*P*^ allow to obtain the activity of regulators at each sample and to detect per sample gene perturbation, respectively. In this section, we aim to leverage these functionalities to capture tumour subtype characteristics, and thus give an overview of one possible direct application of LATNET. For LATNET ^*A*^ and LATNET ^*P*^, we want to respectively capture different behaviours of the regulators activity and perturbed genes that could be specific to tumour subtypes. For both signals as well as for EXPRESSION, we follow the same experimental procedure. We first perform a per-subtype computation of the mean signal for each variable. We then construct a correlation network for the features of each signal. To ease the visualization-based comparison, for each subtype we sort genes by their mean signal across samples and we restrain the visualization on the top-100 features. We uses a unified cut-off of 0.2 such that two nodes in the network are only connected if their correlation is above the threshold. The cut-off was chosen visually (in an interactive way) to increase connectivity between nodes without overwhelming the visualization plot. Figure 3 shows a network visualization of the obtained results on each signal and each subtype.

In Fig. 3, we notice a very clear distinction in terms of the regulators activities in each of the breast cancer subtypes. For the perturbation, the change in signal intensities is lower on different subtypes. Nevertheless, complementary subnetworks are observed between Basal and LumA networks and between Basal-LumA and LumB-Her2 networks. In contrast, for the expression signal it is very hard to find such complementarity between the four networks. Although a per gene/regulator biological study of the results is beyond the scope of this paper, we note that, in contrast to EXPRESSION, i) among the most active regulators, we find multiple transcription factors known to be associated with breast cancer in the literature, including ESR1, E2F2, E2F3, BRCA1, BRCA2, CCNE1 and others [31], ii) we find multiple perturbed genes that have been found to be altered in breast cancer, including PP1RIB, DEFB1, GPR161 and others [32].

## Conclusion

Gene expression data analysis is among the major topics in bioinformatics and computational biology that could rise impactful insights in a wide range of real-world problems, including diagnosis and personalized medicine. With the recent advancements in experimental procedures, huge quantities of expression data are made available, and the need for efficient and large-scale analysis tools capable of providing system level insights is all the more urgent. In multiple gene expression data analyses, existing well-established approaches could perform poorly simply because the raw input signal (expression) could be flat, noisy and not informative. Besides, multiple signals of patterns could be hidden or weakly observed directly in the expression data. In this paper, we proposed LATNET, a signal transformation framework that allows to generate from an initial large-scale gene expression data new latent representations based on a hidden network structure defining relations between the genes. LATNET considers the genes in a systemic perspective and aims to leverage existing background knowledge about the relations between them (i.e., regulatory networks, co-expression networks,...) as an underlying hidden structure to perform signal transformations. We proposed an implementation of LATNET that leverages a gene regulatory network structure between the genes to unravel latent signals expressing the activity level of regulators and the perturbation level of target genes in the given data context. For a practical usage of our framework in real-world applications, we also provided a parallel implementation making it scalable to large-scale input datasets and we showed how LATNET could be used to perform classification and comparative analyses. Experimental results of using LATNET for the analysis of gene expression data of bladder cancer show the efficiency of our framework and how the performances, including stability and reproducibility, are enhanced compared to state-of-the-art latent methods and to the original expression data. Additionally, the case study performed on gene expression data of breast cancer shows the ability of our method to find relevant biomarkers. Lastly, we believe the ability to generate latent sample-specific regulatory signals using hidden network structure will greatly facilitate the application of network-based methods to the increasingly large, complex omics datasets, and ultimately support the emerging field of precision network medicine.

## Abbreviations

AUC: Area under the curve
DE: Differential expression
GO: Gene ontology
GRN: Gene regulatory network
MF: Matrix factorisation
NMF: Non negative matrix factorisation
PCA: Principal component analysis
RF: Random forest
RFE: Recursive feature elimination
SVD: Singular value decomposition
SVM: Support vector machine
TF: Transcription factor
